# Assessing the Feasibility of Using Remote Sensing Data and Vegetation Indices in the Estimation of Land Subject to Consolidation

**DOI:** 10.3390/s24237736

**Published:** 2024-12-03

**Authors:** Katarzyna Kocur-Bera, Anna Małek

**Affiliations:** 1 Institute of Geodesy and Civil Engineering, University of Warmia Mazury in Olsztyn, 10-719 Olsztyn, Poland; 2 Institute of Civil Engineering, Faculty of Civil and Transport Engineering, Poznan University of Technology, 60-965 Poznan, Poland; anna.malek@put.poznan.pl

**Keywords:** land consolidation, UAV multispectral images, *NDVI*, *SAVI*, photogrammetry

## Abstract

The values of vegetation indices can provide a new source of data for use in the estimation of land to be consolidated. The results of research work carried out so far indicate a significant advantage of low-volume imaging over satellite methods when it comes to calculating vegetation index values. This paper analyses multispectral images for the areas of selected croplands acquired via the Sentinel-2 satellite and an unmanned aerial vehicle (UAV) equipped with a multispectral camera. The research work consisted of evaluating *NDVI* (Normalised Difference Vegetation Index) and *SAVI* (Soil Adjusted Vegetation Index) values depending on the type of crop grown, the size of the cultivated area and the method of data acquisition. The data obtained were used to assess their potential use in the estimation of land to be consolidated. The effect of land consolidation is primarily to create more favourable living conditions and increase agricultural productivity. The results of the study showed that it would be preferable to use multispectral images acquired using UAVs rather than those from Sentinel satellites. This is due to the insufficient resolution of the satellite data, the correlation of *NDVI* and *SAVI* values at only a satisfactory level and the low accuracy of the data obtained for small registered plots of land.

## 1. Introduction

### 1.1. Background

Land consolidation is an administrative and technical process that reorganises the ownership of agricultural land (registered plots) in order to increase the efficiency of agricultural production, optimise land use and improve living conditions in rural areas. Consolidations are carried out by exchanging, combining or dividing registered plots of land, which, in this way, create more compact, regular areas. In terms of environmental protection, the land consolidation process can lead to better management of natural resources such as water and soil, which contributes to environmental protection and multifunctional sustainable rural development. As pointed out in publication [1], there is a need for an interdisciplinary approach in the land consolidation process, to take into account the aspects of spatial planning, environmental protection, economic and landscape values. The implementation of land ownership reorganisation should follow the principles of modern sustainable agriculture, i.e., fulfilling production, economic, ecological and social objectives simultaneously [2]. Maintaining these objectives is a condition for the coherence and mutual order of amalgamation processes.

### 1.2. Agri-Environmental Factors in the Land Consolidation Process

In contrast to practices used in many European Union countries, where great attention is paid to the ecological development of the countryside, Polish legislation does not take into account a number of other aspects of consolidation works, apart from their role in land concentration and improvement of the agrarian structure [3]. In the spirit of sustainable development, land use and ownership activities should take into account economic, social, economic and ecological aspects. For this reason, the assumptions made for the implementation of scale-ups should reflect the above factors. According to the authors of paper [4], attention should be paid to the adopted pan-European agricultural model, focused on the protection and preservation of the natural and landscape values of the countryside, together with an increase in profitability in the agricultural sector. The authors of this article point to the multitude of agri-environmental programmes that support the preservation and development of biodiversity and, at the same time, rural development. The beneficial aspect of being able to obtain certain subsidies is taken into account in land appraisal by, among others, Danish scale planners [5]. The European Union’s rural policy focuses on the diversification of the functions of these areas through, among other things, Rural Development Programmes (RDPs). As the authors of publication [6] point out, in order to meet the requirements of sustainable development, the following factors should be taken into account during consolidation work: criteria for the selection of environmental factors; methods for assessing the sustainability of agriculture; environmental conditions related to the variability of weather conditions, water and soil quality; factors affecting the efficiency of agricultural production; and infrastructural issues such as the condition and distribution of roads and water drainage. This coincides with the list of agri-environmental indicators produced by the Organisation for Economic Co-operation and Development (OECD), which are applicable to integration work. Also, the Food and Agriculture Organisation of the United Nations (FAO) recommends that state institutions carry out activities to support projects that protect the environment and climate and shape biodiversity during land consolidation processes [7]. The reorganisation of agricultural plots can, among other things, reduce CO_2_ emissions as a result of the reduction in fuel consumption needed during transport and the cultivation of agricultural land [8]. As the authors of publication [9] note, the way land is used, its resource requirements and landscape management practices are linked to many environmental and climate-related challenges. In countries with a longer tradition and knowledge (experience) of scale-ups, like Denmark, the Netherlands or Finland, agri-environmental factors are taken into account to a large extent when planning a new land distribution. The land appraisal (valuation) process itself poses challenges in terms of the availability of information to maintain the principle of equivalence and ‘at least as well off’ [10]. The Danish model, for example, is based on indexing a number of agri-environmental factors, such as soil quality, agricultural potential, drainage, opportunities for certain subsidies, etc., which together give a maximum value of 100 [5]. In the Netherlands, on the other hand, land valuation is mainly based on the productive capacity of the farm, for which soil maps and groundwater level maps are the basis. Factors related to the shape of the plot or its accessibility are not taken into account [11]. Similarly, in Germany, the actual benefits that a plot of land can bring are taken into account, regardless of the distance from the farm or village centre [12]. In Turkey, on the other hand, soil conditions, determined by mapping and yield potential, account for 60% of the score awarded during the valuation of land undergoing the scale-up process [13]. There are also countries, such as Lithuania (which is a post-Soviet country), in which researchers [14] note the occurrence of statistically significant changes in spatial patterns of land use and land distribution since the 1970s, which calls for explicit policies related to land management and use.

### 1.3. Techniques for Obtaining Agri-Environmental Data

The development of new technologies offers the opportunity to use increasingly precise methods of research data acquisition and analysis. As the authors of publication [15] note, current methods in land valuation in many countries lag behind existing techniques and technologies. The values of the vegetation indices *NDVI* (Normalised Difference Vegetation Index) and *SAVI* (Soil Adjusted Vegetation Index) can be new sources of data used in the estimation of land subject to consolidation. They provide important information on the state of vegetation, the density of vegetation cover, the level of irrigation or the quality of cultivation techniques, which can be a timely and accurate source of data to be taken into account in the estimation of land to be consolidated. Currently, the development of a comparative land appraisal mainly takes into account the quality of the land and the agricultural suitability of the soil based on geodetic and cartographic studies, such as cadastral and classification maps. As the authors of [16] point out, the key criterion for valuation is agronomic value from the farmer’s point of view. The use of vegetation indices can assist in the process of properly assessing the agricultural suitability of land, as they are a direct reflection of the prevailing conditions in the context of agricultural croplands and yield potential. Remote sensing data acquired at the land appraisal stage can provide the most up-to-date source of data to be taken into account when assessing the agricultural suitability of land. Studying the literature on the subject, it was noted that the values for vegetation indices can be calculated from satellite images from, for example, the European Space Agency’s Sentinel 2A and Sentinel 2B satellites, or the Terra environmental satellite. The spatial resolution of the images acquired in this way is, for example, 10 m by 10 m for a single pixel. The results of previous research work [17,18,19,20,21,22] indicate significant advantages of low-altitude imaging (UAV) over satellite-based methods, including lower costs, flexibility in data acquisition through freedom of choice of the survey date and area, higher resolution of the acquired images, and, globally, lower weight and dimensions. Also in favour of the use of UAVs is the possibility of using different measurement sensors. Equipping the UAV with a multispectral, thermal imaging and classic RGB camera offers the possibility of monitoring the environment over a wide range [23]. This can be used to obtain data on plant diseases [24] and weed incidence [25], and to determine biomass and estimate yields [26], as well as to determine water stress and increase irrigation needs [27,28]. In addition, appropriate longitudinal and transverse coverage of the images can be designed each time, depending on the needs of the study, and a trajectory can be planned, including in an automated process. Most unmanned aerial vehicles (UAVs) are additionally equipped with a GNSS receiver, which provides the opportunity to create a precise orthophotomap, terrain and object model product. The practical use of UAVs in surveying for, among other things, object inventory and cadastral work is presented in papers [29,30].

## 2. Materials and Methods

### 2.1. Research Concept

The factors presented in the Introduction section led the authors to carry out research aimed at assessing the values of the *NDVI* and *SAVI* vegetation indices depending on the type of crop grown, cropland area, and the method of data acquisition (satellite versus UAV) in the context of their subsequent use in the land estimation process for scale-ups.

**Figure 1:**
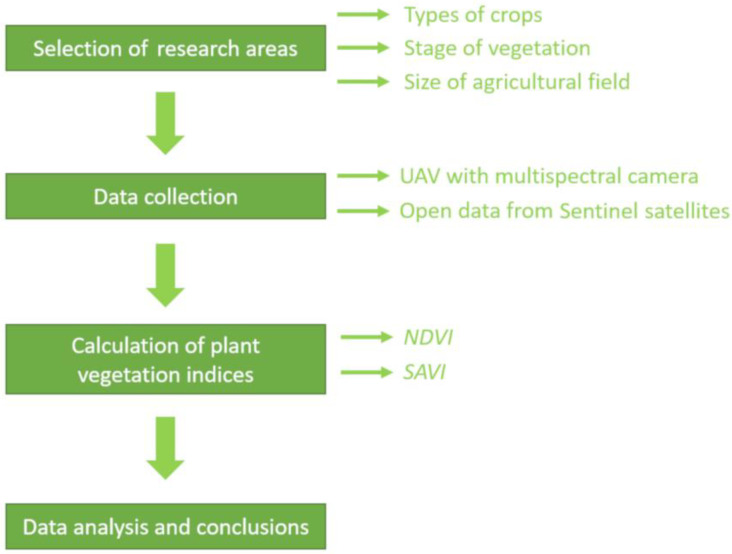
Flow chart of the research work carried out.

The study was planned and carried out according to the scheme shown in Figure 1. In the initial phase, survey areas (agricultural land with different crops and in different stages of growth) were selected and flights using a UAV with a multispectral camera (at different altitudes) were planned and carried out in a timeframe compatible with the possibility of acquiring data from Sentinel satellites (the satellite data timeline was 5 days). In the next step, the values of the *NDVI* and *SAVI* plant vegetation indices were calculated using data from unmanned aerial vehicle flights at five different altitudes and Sentinel satellites. In the final stage of the research, an analysis of the data obtained was carried out and conclusions were drawn.

**Figure 2:**
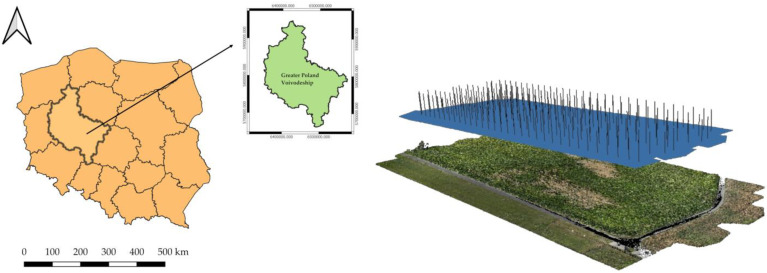
Location of the study area and an example of the imaged area in the form of a maize field represented as a point cloud.

The study area is located in central-western Poland, in the Wielkopolska Province (Figure 2). According to the Central Statistical Office [31], the geodetic area of the province is 2982.7 thousand ha, of which agricultural land accounts for 64.5% (national average 59.9%). The soils in the area under study developed mainly from post-glacial sediments, being sandy and clayey formations. The soils are predominantly light and very light, mainly podzols and brown soils. Arable soils, which are the predominant land use in the Wielkopolska Province, are classified primarily as low-fertility and low-productivity, too light and too dry, hence yields are closely dependent on the prevailing weather conditions. At the same time, the area is among those with the lowest annual rainfall, averaging 500–600 mm. Periodic droughts are observed during critical parts of the plant growing season—mainly in spring, late April/early May, and late autumn, as early as October [32]. The existing water deficit, which directly affects the quantity and quality of agricultural yields, is proposed to be improved by means of the development of small-scale retention in rural areas [33].

There are a number of different vegetation indices [34,35], which, when calculated and presented on a map, provide important information on vegetation condition, vegetation cover density or irrigation levels. To calculate them, it is necessary to acquire data in different bands (spectra). For example, the *NDVI* is treated as a general indicator of plant health, as its value is directly related to the chlorophyll content of the plant [36]. Chlorophyll, which is involved in photosynthesis and responds to different types of stress factors, manifests itself in different colourings of the plant leaf [37]. Different plant species (and therefore crops) are characterised by different growth stages, structures or sowing densities. Near-infrared reflectance and absorption of red radiation are time-varying and depend on the content of photosynthetically active pigments [38]. For this reason, the research results presented in this article assume crops at different stages of growth, with different sowing densities and current compactness. The study area was a field of maize, buckwheat, celery and rapeseed.

### 2.2. UAV-Based Data Collection

As part of the research, imagery was acquired by remote sensing using a DJI Mavic 3 unmanned aerial vehicle equipped with a 4/3-inch CMOS RGB camera and a multispectral camera. The RGB sensor mounted on the device can take 20 MP JPEG photos, while the multispectral sensor can take 5 MP photos in TIFF format. The cameras are integrated on a three-axis mechanical stabilisation system. The maximum image size is 5280 × 3956 pixels (RGB) or 2592 × 1944 pixels (multispectral), and the focal length equivalent is 24 mm. The monochrome sensors take images in green (G 560 ± 16 nm), red (R 650 ± 16 nm), red edge (RE 730 ± 16 nm) and near-infrared (NIR 860 ± 26 nm) channels. The unmanned aircraft used in the study is also equipped with a precision positioning system (GNSS), using position corrections from reference stations (using corrections from the ASG-EUPOS system) via a mounted RTK receiver. This allows an accuracy of ±2 cm situationally and ±5 cm altitudinally in determining the centre coordinates of each of the images taken. With this solution, comparable accuracy of the resulting orthophotomaps is achieved, as with the use of photopoints and a classic GNSS receiver [39].

Field survey work was carried out during the month of July 2024, selecting those days on which Sentinel-2 satellite imagery was also acquired for the study area. Research missions using the UAV were planned in an automated manner. The transverse and longitudinal coverage of the images was 70% and the camera was oriented in the nadir position. Images were acquired at several heights—10 m, 20 m, 30 m, 50 m and 100 m.

### 2.3. Satellite-Based Data Collection

In 2015 and 2017, with the cooperation of the European Space Agency (ESA) and the European Union (EU), Sentinel-2 mission satellites [40] equipped with multispectral sensors were sent into Earth orbit, with the main purpose of monitoring the Earth’s land cover and land use and ongoing climate change. The sensors acquire images in a range of bands, from visible to shortwave infrared, with resolutions of 10 m, 20 m and 60 m. In total, images are acquired in twelve bands of varying length. The performance of the acquired data in various applications is described by the authors of publication [41], who point out the fairly high spatial (10 m) and temporal (5 days) resolution and the availability of red edge bands as the main advantages. In publication [42], the authors highlight the open access to the images acquired by the Sentinel-2 satellites and the free access to software allowing their processing, which offers the possibility of their subsequent wide use in precision agriculture. The main areas of application include monitoring crop health [43], predicting fertiliser and pesticide use, or detecting stress caused by limited access to water [44]. In this article, the research area data used were obtained from the European Space Agency (ESA) website [45]. Near-infrared (842 nm) and red (665 nm) images were taken.

### 2.4. Vegetation Indexes

Devices in the form of electromagnetic radiation sensors record waves reflected from different types of surfaces. Among other things, they can be used to assess the condition of plants. The amount of radiation absorbed and reflected from a given plant species depends on its condition (health) and phenological phase [46,47]. Among the most commonly used vegetation indices (*VI*) in precision agriculture is the Normalised Difference Vegetation Index (*NDVI*). Its value allows, above all, to distinguish between areas covered by vegetation and other (artificial) types of land cover. Visualisation on the map indicates abnormalities in the plant growth process. The index value is obtained by comparing the amount of absorbed and reflected radiation in the red and near-infrared bands according to Equation (1) [48,49]:

*NDVI* = (NIR − Red)/(NIR + Red) (1)

where

NIR—near-infrared channel reflectance,

Red—red channel reflectance.

The higher the index value, the healthier the plant. The value of the index ranges from −1 to 1, with values close to −1 defining water-covered areas and those between −0.1 and 0.1 indicating bare soil (no vegetation cover). *NDVI* values above 0.6 are considered an indicator of vegetation with high vitality, and values close to 1 characterise plants at the highest stage of development and in excellent health [50]. Studies on vegetation indices [51,52] have shown that parameters such as soil colour, moisture and, above all, plant densities shape the value of the *NDVI*; hence, for areas with low vegetation cover densities, the Soil Adjusted Vegetation Index (*SAVI*), calculated according to Equation (2) [53], is more commonly used. The value of the index is calculated similarly to the *NDVI* based on the same spectral ranges, but an additional soil parameter, L, is introduced, taking values from 0–1, depending on the density of the vegetation cover, with higher values indicating greater density. The outcome values of the *SAVI* are between −1.0 and 1.0 [54].

*SAVI* = {(NIR − Red)/(NIR+ Red + L)} * (1 + L) (2)

where

L—soil parameter: L = 1.0 is adopted for areas without vegetation cover, L = 0.5 for moderate-density vegetation cover and L = 0.0 for high-density vegetation cover (equivalent to *NDVI*).

**Figure 3:**
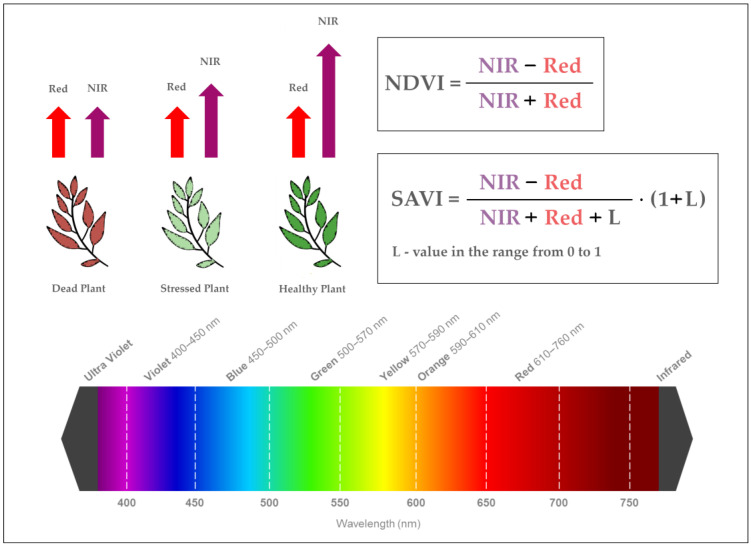
Method of calculating *NDVI* and *SAVI* values with interpretation of plant condition.

Figure 3 shows a graphical interpretation of the plant condition in relation to how the *NDVI* and *SAVI* plant vegetation index values were calculated. In this study, a value of L = 0.5 was used in the calculation of the *SAVI*, which results in a reduction in the presence of soil noise with different crop types and compactness [55].

### 2.5. Image Processing Methods

**Figure 4:**
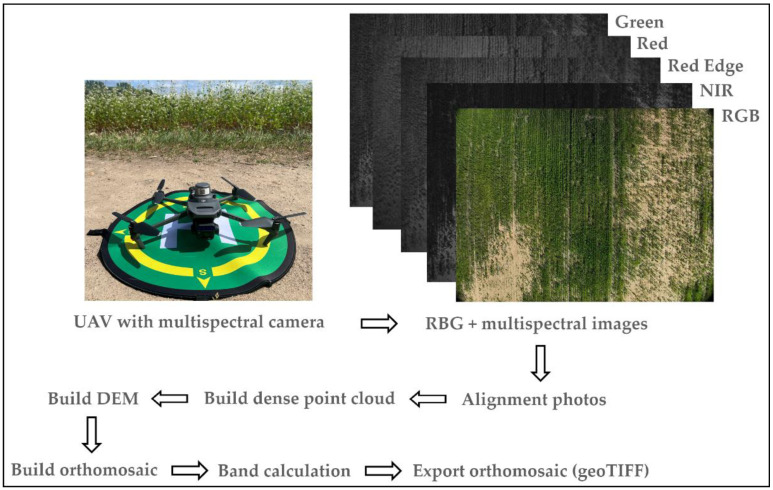
Diagram describing the process of creating an orthophotomap.

For each study area, a process leading to an orthophotomap was carried out in Agisoft Metashape. This process is schematically illustrated in Figure 4. Once the TIFF-format images were uploaded, an alignment of the block of images was performed, followed by camera optimisation (to increase accuracy), after which a dense point cloud and numerical terrain model were built. Lastly, an orthophotomap was created. The output product was exported in geoTIFF format (uncompressed), 16 bit, for each channel (band) separately (green—G, red edge—RE, near-infrared radiation—NIR). The calculations leading to the values of vegetation indices (*VI*) *NDVI* and *SAVI* were carried out in QGIS software ver. 3.34.12.

### 2.6. Experimental Site

At the planning stage of a new land distribution/layout in different countries [4,10], factors related to soil quality, the level of agricultural culture and the productivity of the farm are of varying importance. This also applies to the sources of data used and timeliness. The physical and chemical properties of the soil affect its ability to hold and provide nutrients to plants and depend on a number of factors, such as water availability, temperature and insolation, the type of crop or the agrotechnical techniques used. Therefore, depending on the productivity of the farm, its economic efficiency within the same land class can vary. As presented by the author of [56], the structure of production and its value at a given time on a farm depends primarily on the land resources, i.e., the quality of the land, its distribution or its use. For this reason, the *NDVI* [57] was used in this study to assess the productivity of agricultural plots of land. This indicator was first used in 1973, so it is both the oldest and best-known indicator for describing the vegetation characteristics of plants and, due to its ease of calculation, the most widely used. It describes the developmental status of the plants and their health (vigour). Its value is influenced by a number of factors, including the effect of noise from the soil showing through vegetation. For this reason, coefficients have been developed to minimise this impact, e.g., the *SAVI* [53], which was also used in this study.

In this study, different species with different density levels and in different vegetation phases were used to identify differences in the reflection and absorption of radiation by the plants. The analyses included the following plant species:

- Maize in the middle stage of growth, with numerous areas deprived of seedlings due to the prevailing water and soil conditions;
- Celery, planted as a row crop in the middle growth phase;
- Rapeseed with high compactness, just before harvest;
- Buckwheat in the early growth phase.

In the analyses carried out as part of this study, attention was paid to the shape and size of the registered plots in the areas subject to consolidation processes. This has been the subject of much research work, and the results indicate that the area of a significant proportion of these plots is less than 1 hectare [58,59,60]. The shape and size of plots of land has a huge impact on the profitability of agricultural production. Depending on the geometric parameters of the plot, the variation in cultivation costs can be as high as €1400/year/ha [61]. Within the crops analysed, several reference plots were adopted for each crop, representing study areas that ranged from 0.17 ha to 4.70 ha. The crop lands selected for the research were characterized by a uniform soil class.

## 3. Results

In the first stage of this study, airstrikes were carried out with an unmanned aerial vehicle recording multispectral images for the selected study areas (agricultural plots) from five different heights: 10 m, 20 m, 30 m, 50 m and 100 m. Detailed data for one of the study areas are presented in Table 1. Analysis of the calculated values of the *NDVI* and *SAVI* vegetation indices for the study areas showed that the height of the flight carried out had no effect on the values obtained, with an assumed threshold of accuracy of two significant digits (for example: *NDVI*/*SAVI* values for maize crop at an altitude of 10 m to 30 m—0.5001/0.4906, 50 m—0.5010/0.4914, 100 m—0.4955/0.4922). Therefore, only data from the UAV flight at a 100 m ceiling, for which the Ground Sample Distance (GSD) was 4.6 cm/px, were used in further analyses. As can be seen from the analysis of the data summarised in Table 1, this resulted in more than 6 times shorter flight times and more than 10 times fewer multispectral images acquired (which translates into data file sizes) when comparing measurements taken at 10 m and 100 m. Each of the analysed crops was divided into study areas as follows:

- Buckwheat into three, with areas of 14,437 m^2^, 8903 m^2^ and 5534 m^2^;
- Rapeseed into three, with areas of 9710 m^2^, 6160 m^2^ and 3550 m^2^;
- Celery into three, with areas of 5380 m^2^, 3690 m^2^ and 1690 m^2^;
- Maize into four, with areas of 46,950 m^2^, 24200 m^2^, 13,150 m^2^ and 9600 m^2^.

**Figure 5:**
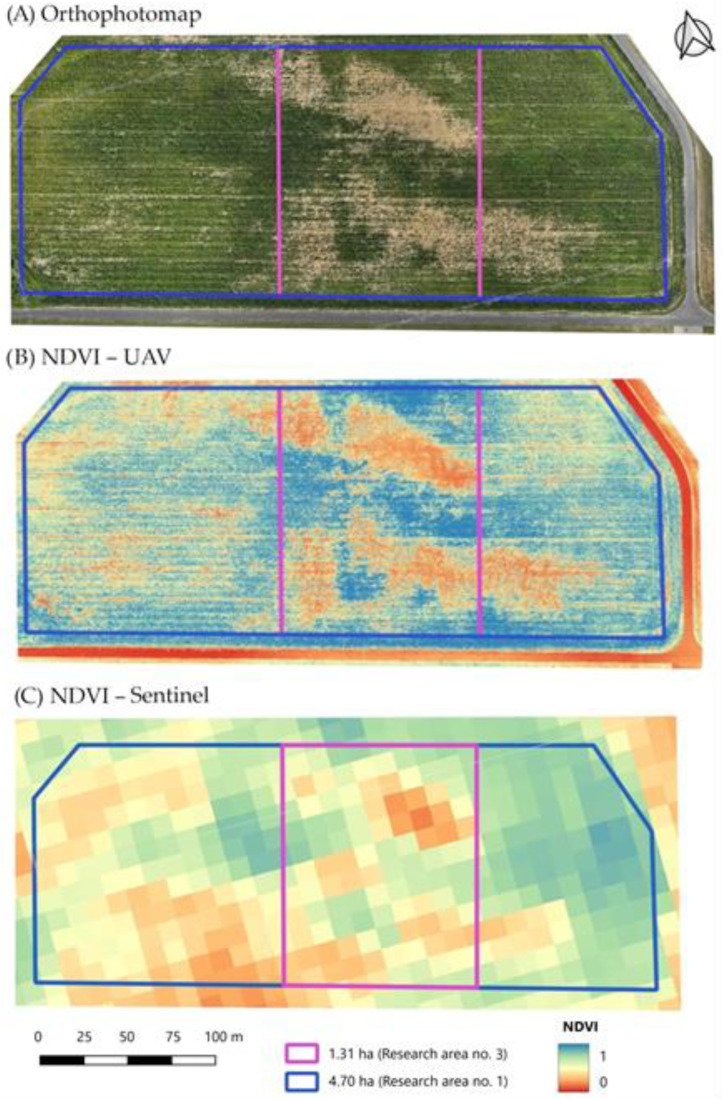
Research areas with maize cultivation as orthophotomaps (**A**), and visualisation of *NDVI* values based on images acquired from UAVs (**B**) and Sentinel satellites (**C**).

An example of a research area in the form of a maize-growing plot of land is shown in Figure 5. In this study, analyses were carried out for the entire cultivation area (representing the research area—numbered 1) and after dividing it into two or three smaller parts (representing the subsequent research areas—numbered 2, 3 and possibly 4, where the area decreases as the number increases).

**Figure 6:**
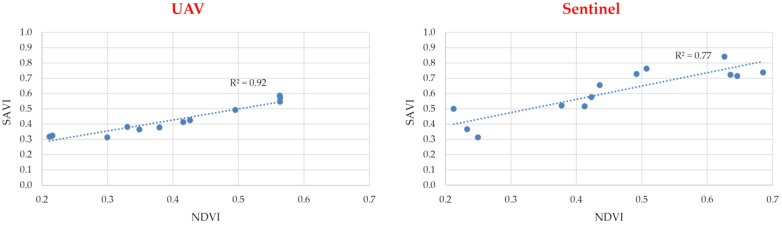
Correlation between *SAVI* and *NDVI* vegetation indices calculated from multispectral images acquired from UAVs and Sentinel satellites.

For each of the study areas, graphs were produced showing the correlation between the calculated *NDVI* and *SAVI* values (Figure 6), based on data acquired from the UAV and satellite (Sentinel satellites) levels. The *NDVI* and *SAVI* vegetation index values calculated for each study area are summarised in Table 2 and Table 3. The correlation of *SAVI* and *NDVI* was assessed based on the value of the *R*^2^ coefficient of determination. The higher the value of the *SAVI*, the greater the occurrence of vegetation cover on the ground. On the other hand, the higher the *NDVI* value, the higher the chlorophyll content of the plant, so the better its health and physical condition/vegetation. Given that the *SAVI* is a modification of the *NDVI*, the correlation between *SAVI* and *NDVI* values should be very strong. This is confirmed by the studies presented in paper [62,63]. In the present study, for the UAV-acquired data, this correlation was found to be strong, with *R*^2^ = 0.92; and for the Sentinel satellite-derived data, it was found to be satisfactory, with *R*^2^ = 0.77. In the case of the images acquired from the Sentinel satellites, the correlation between the indicators is significantly lower, so the values of the individual indicators describing farm productivity are not as meaningful as in the case of UAV use. This is related to the pixel size, which is 10 m by 10 m, and the averaging of the data from such an area. Therefore, when carrying out analyses using *NDVI* and *SAVI* vegetation index values simultaneously, UAV-acquired data should be used due to the importance of more accurate data. A graphical interpretation of the *NDVI* values for the selected study area is shown in Figure 5.

**Figure 7:**
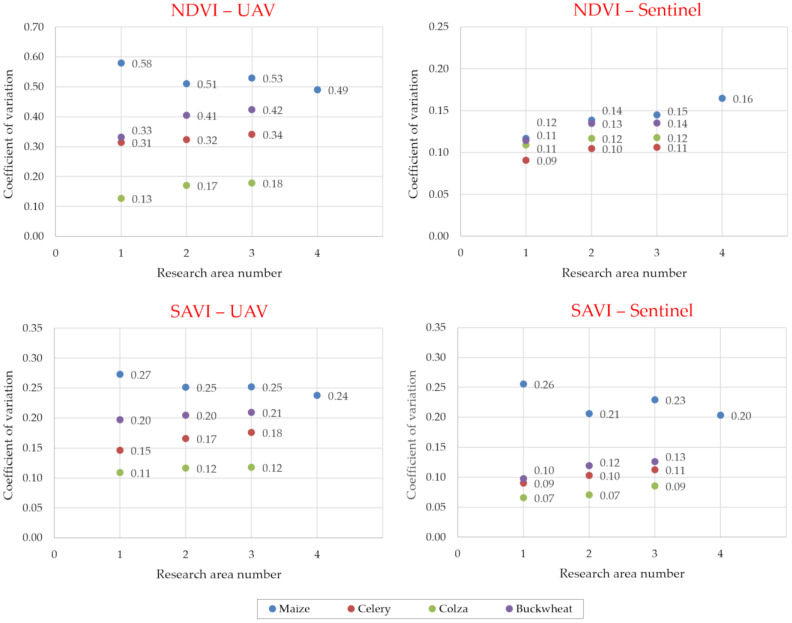
Values of the coefficient of variation for *NDVI* and *SAVI* indices, calculated from multispectral images acquired from UAVs and Sentinel satellites.

In order to determine how much the values of *NDVI* and *SAVI* varied within a given crop (study area) using the data acquired with the UAVs and Sentinel satellites, the values of the coefficient of variation (the quotient of the standard deviation value and the mean value of the measurements) were calculated. The results obtained are presented as graphs in Figure 7.

The coefficient of variation takes values from 0 to 1, where the higher the value, the more diverse (heterogeneous) the study area. Analysis of the data shown in Figure 7 reveals that that for the multispectral images acquired with an unmanned aerial vehicle, the variation in the *NDVI* values from the mean value for the crop and study area is up to about four times greater than for the data acquired with Sentinel satellites (the maximum difference is 0.42). The values of the coefficient of variation for the *NDVI* are similar (maximum difference value of 0.06) for the UAV- and Sentinel-satellite-derived results only for rapeseed with high compactness, just before harvest. This may be due to the characteristics (appearance) of rapeseed in its final stage of vegetation, and its high density in the cropland area. When comparing the results of data acquired with UAVs and Sentinel satellites, it can be noted that the variation in the coefficient of variation values for the *SAVI* is much smaller than for the *NDVI* (the maximum difference is equal to 0.10). It can be observed that in the case of maize (in the middle phase of growth, with numerous areas deprived of seedlings), there is a large discrepancy between the *NDVI* and *SAVI* values for both methods of data acquisition. For example, for the entire study area of 4.70 ha, the *NDVI* based on the UAV data is 0.43, while the *SAVI* is equal to 0.42. In contrast, for the study area of 1.31 ha, where there are numerous thinnings, both the *NDVI* and *SAVI* have a value of 0.38. In the case of the Sentinel satellite data, these are, respectively, for the 4.70 ha area, values of 0.42 and 0.38 for the *NDVI* indicator, and for the 1.31 ha area, values of 0.58 and 0.52 for the *SAVI* indicator. With a field pixel size of 10 m, the satellite data, and therefore the values of the vegetation indices, are averaged and do not accurately capture local variations in plant density caused by water and soil conditions, especially for smaller plots. This is evident in the analysis of the data for the buckwheat crop (Table 2 and Table 3). The *NDVI* and *SAVI* values for all the study areas (i.e., no. 1 with a surface area of 1.44 ha, no. 2 with a surface area of 0.89 ha and 0.55 ha) are 0.21–0.22 and 0.32 for the UAV-acquired data, respectively. In contrast, using the multispectral images from the Sentinel satellites, the *NDVI* and *SAVI* values for the analogous study areas are 0.51–0.63 and 0.65–0.84, respectively.

## 4. Discussion

An analysis of the advantages and applicability of multispectral cameras placed on UAVs was carried out by, among others, the authors of paper [64]. In their conclusions, they pointed to the rapid acquisition of accurate information and the low cost of purchasing equipment and performing tests. They also stressed the high importance of such analyses in the context of sustainable development and environmental protection. An increasing number of authors are supporting UAVs as a tool to support integration processes, mainly in low-altitude photogrammetric studies [65,66,67]. The vegetation indices developed on the basis of multispectral imaging can be linked to environmental, economic and social factors, which can then be taken into account in the estimation of land subject to consolidation processes. In this study, analyses were carried out to assess the feasibility of using free data acquired from Sentinel satellites and data collected with the aforementioned UAV equipped with a multispectral camera for this purpose. In this case, it should be mentioned that the spatial resolution of satellite imagery (determined by pixel size) is, in the case of the Sentinel satellite data, 10 m. This means that an average reflectance value is calculated from a 10 m × 10 m area, and thus is a vegetation index value. In addition, weather conditions (cloud cover), in the case of satellite imagery and the temporal resolution of the acquisition, have an impact on the acquired data. The advantage of this method is that data can be acquired for large areas in a short period of time. In the case of multispectral image acquisition using a UAV, analyses of the collected data have shown that at a flight height of 100 m, an accuracy of *NDVI* and *SAVI* values of two significant digits is obtained, the same as for a flight height of, say, 10 m. The results carried out and presented within this publication show that data can be acquired for an area of approximately 1 ha in about 5 min.

The results of the analyses presented in Figure 6 and Figure 7 and Table 2 and Table 3 confirm the observations of the authors of papers [17,18,19,20,21,22] on the significant advantages of low-probe imaging over the satellite method (in this case Sentinel satellites) when assessing plant vegetation indices. It was even observed that the variation in the coefficient of variation values for the *NDVI* was about four times greater when using data from the UAV method compared to data from the Sentinel satellites. The *R*^2^ coefficient of determination, describing the correlation between the *SAVI* and *NDVI* values for the data acquired using the UAV, was much closer to unity, at *R*^2^ = 0.92, compared to the *R*^2^ = 0.77 determined for the data obtained from Sentinel satellite imagery. The results of the study showed clear differences in the accuracy of the acquired data using the satellite method, especially for small areas (registered plots of land).

One of the basic methods of carrying out land estimation for the purpose of land consolidation works in Poland is the Wrzochoł–Dawidziuk index method [68]. This method is based on the qualitative classification and agricultural suitability of the land, taking into account the relationship between the land class and its production value [69]. The values of the conversion coefficients are then selected on the basis of the quality class and the agricultural land suitability complex, ranging from 5 to 100, where the lowest value is for wasteland and the highest value is for the highest-quality class and the highest agricultural land suitability complex (best). The main data used are those derived from cadastral and soil-agricultural maps. The method presented in this paper for the use of low-altitude multispectral imaging (UAV) data represents a novelty when it comes to its use in the land estimation process for land consolidation work. The use of plant vegetation indices (in this case, the *NDVI* and *SAVI*) in this area would make it possible to realistically determine the production capacity of a given plot (farm), and not just on the basis of map data. Given that the pilot analyses used a variety of species at different densities and vegetation phases, as well as different cultivation areas (including the simulation of small plots with crops), it can be considered that, with the research model adopted in this study, the UAV method can be taken into account in the estimation of land undergoing consolidation. In this originally adopted model, it is planned to add an additional criterion, related to the production parameters of a given farm (cropland) on the basis of the value of the plant vegetation index, to the Wrzochół–Dawidziuk index method used in Poland [68]. In the method proposed by these authors, points ranging from 0 to 100 would be awarded for a given registered plot depending on the value of the vegetation coefficient. Given that *NDVI* or *SAVI* values range from −1 to 1, and that values closer to 1 are the most desirable (plant in best condition/vegetation), the point value would be the equivalent of a positive vegetation index value multiplied by 100. In further research, it is planned to acquire data at different vegetation stages for different plant species, and to check the correlation between the remote sensing data (from the UAV) and the ‘in situ’ data measured with the chlorophyll meter. On this basis, it is planned to determine which vegetation indicator would best reflect the productivity of the farm. It is also planned to carry out research into the existence of correlations between the values of vegetation indices and the land class and agricultural land suitability complex. The final result of this work would be to propose a method for carrying out an estimation of land to be consolidated using vegetation indices. Table 4 and Table 5 (based on the data presented in Table 2 and Table 3) show the results of calculating a coefficient for a criterion related to the productivity of a habitat—tentatively named HP (Habitat Productivity). The values of the *HP*_NDVI_ coefficient represent the value of the *NDVI* vegetation index multiplied by 100, and those of the *HP*_SAVI_ analogously for the *SAVI* vegetation index. Analysing the data presented in Table 4 and Table 5, the following observations can be made:

- For maize depending on the area of the crop land (in the research area), the difference in the values of the *HP*_NDVI_ coefficient is up to 12 points when using UAV data and 11 points for Sentinel data; for the *HP*_SAVI_ coefficient in a similar way, the values are 11 points for UAV and 19 points for satellite data.
- In the case of celery depending on the area of crop land (in the research area), the values of the *HP*_NDVI_ coefficient are identical for data acquisition with UAVs and differ by 5 points for data from a Sentinel; for the *HP*_SAVI_ coefficient, the difference in values is 4 points for UAVs and 3 points for satellite data.
- For colza, depending on the area of the crop land (in the research area), the difference in the values of the *HP*_NDVI_ coefficient is up to 5 points in the case of UAV data and 4 points when using data from a Sentinel; for the *HP*_SAVI_ coefficient, in a similar way, the values are 8 points for UAV and 16 points for satellite data.
- In the case of buckwheat, depending on the area of the crop land (in research area), the difference in the values of the *HP*_NDVI_ coefficient is up to 1 point in the case of UAV data, and up to 19 points when using satellite data; for the *HP*_SAVI_ coefficient, in an analogous way, the values are identical for UAV data and differ by 19 points for Sentinel satellite data. The maximum differences in the scoring of crop lands for the purpose of the estimation of land subject to consolidation, using the *HP*_NDVI_ and HPSAVI coefficients, are up to 11 points for UAV data and up to 19 points for Sentinel satellite data. As can be seen from the above, with the same soil class and uniformity in the conduct of agrotechnical treatments, the differences in *HP*_NDVI_ and *HP*_SAVI_ values for a crop can be significant. The determined values of the *HP*_NDVI_ coefficient for the maize based on the UAV and Sentinel data are almost identical (the maximum difference is 1 point). However, in the case of buckwheat, the difference is significant, ranging from 22 to 42 points depending on the area of crop land. Analysing the values of the *HP*_SAVI_ coefficient on the basis of data from UAVs and Sentinel satellites, it is noted that depending on the area of crop land and measurement method, the differences in its values range from 1 to 9 points for colza and from 33 to 52 points for buckwheat. This confirms the influence of the resolution of the acquired data on the final accuracy of the obtained results. The undertaken research indicates the relevance of using data on habitat productivity in the process of estimating land subject to consolidation.

## 5. Conclusions

The following observations can be made from the research and analysis carried out:

- The use of open image data from Sentinel satellites in the estimation of land undergoing consolidation may result in an inaccurate assessment of the production potential of a given plot, due to the insufficient resolution of these 10 m × 10 m images.
- In the case of buckwheat, it was reported that the differences in the values for the data acquisition with UAVs in relation to Sentinel satellites ranged from 0.22 to 0.42 for the NDVI values, 0.33 to 0.52 for the SAVI values.
- A UAV flight at 100 m altitude enables *NDVI* and *SAVI* values to be accurate to two significant digits, the same as a flight at, say, 10 m altitude, resulting in a flight time more than 6 times shorter, and more than 10 times fewer multispectral images acquired.
- For UAV-derived data, the correlation of *NDVI* and *SAVI* vegetation index values was found to be strong, at *R*^2^ = 0.92, and for Sentinel satellite-derived data, it was found to be satisfactory, at *R*^2^ = 0.77.
- For small areas (registered plots), clear differences were noted in the accuracy of the acquired data using the satellite method (Sentinel satellites) compared to the UAV method.
- In this research, the differences in the assessment of the productivity of a habitat (crop) with the same soil class, and uniformity under the conduct of agrotechnical treatments, were up to 10% when using UAV data in the analysis, and up to 20% when using Sentinel satellite data.
- The use of low-altitude multispectral imaging (UAV) data can be a useful tool for assessing the productivity of a plot of crop land during the estimation of land subject to consolidation, and can ultimately improve the economic conditions of farms.

## Figures and Tables

**Table 1:** Detailed data related to the acquisition of multispectral imagery using UAVs for one of the research areas.

No.	Altitude Above the Ground [m]	Flight Time	Number of Photos Taken [pcs]	Ground Sample Distance [cm/px]
1	10	29 min 17 s	584	0.46
2	20	16 min 55 s	312	0.92
3	30	14 min 30 s	204	1.38
4	50	11 min 59 s	117	2.30
5	100	4 min 40 s	57	4.60

**Table 2:** *NDVI* and *SAVI* values calculated for the study areas, analysed on the basis of mulitspectral images acquired with the UAV.

Cultivation	Research Area No. 1			Research Area No. 2			Research Area No. 3			Research Area No. 4		
	Area [ha]	*NDVI*	*SAVI*	Area [ha]	*NDVI*	*SAVI*	Area [ha]	*NDVI*	*SAVI*	Area [ha]	*NDVI*	*SAVI*
Maize	4.70	0.43	0.42	2.42	0.42	0.41	1.31	0.38	0.38	0.96	0.50	0.49
Celery	0.54	0.56	0.55	0.37	0.56	0.59	0.17	0.56	0.57			
Rapeseed	0.97	0.33	0.38	0.62	0.35	0.36	0.36	0.30	0.31			
Cereal *	1.44	0.21	0.32	0.89	0.21	0.32	0.55	0.22	0.32			

* Buckwheat.

**Table 3:** *NDVI* and *SAVI* values calculated for the study areas, analysed on the basis of mulitspectral images acquired with Sentinel satellites.

Cultivation	Research Area No. 1			Research Area No. 2			Research Area No. 3			Research Area No. 4		
	Area [ha]	*NDVI*	*SAVI*	Area [ha]	*NDVI*	*SAVI*	Area [ha]	*NDVI*	*SAVI*	Area [ha]	*NDVI*	*SAVI*
Maize	4.70	0.42	0.58	2.42	0.41	0.52	1.31	0.38	0.52	0.96	0.49	0.73
Celery	0.54	0.65	0.71	0.37	0.64	0.72	0.17	0.69	0.74			
Rapeseed	0.97	0.23	0.37	0.62	0.25	0.31	0.36	0.21	0.50			
Cereal *	1.44	0.51	0.76	0.89	0.63	0.84	0.55	0.44	0.65			

* Buckwheat.

**Table 4:** *HP*_NDVI_ values based on mulitspectral images acquired with UAVs and Sentinel satellites.

Acquisition	Maize		Celery		Colza		Cereal *	
	Area [ha]	*HP*_NDVI_	Area [ha]	*HP*_NDVI_	Area [ha]	*HP*_NDVI_	Area [ha]	*HP*_NDVI_
UAV	4.70	43	0.54	56	0.97	33	1.44	21
Sentinel	4.70	42	0.54	65	0.97	23	1.44	51
UAV	2.42	42	0.37	56	0.62	35	0.89	21
Sentinel	2.42	41	0.37	64	0.62	25	0.89	63
UAV	1.31	38	0.17	56	0.36	30	0.55	22
Sentinel	1.31	38	0.17	69	0.36	21	0.55	44
UAV	0.96	50						
Sentinel	0.96	49						

* Buckwheat.

**Table 5:** *HP*_SAVI_ values based on mulitspectral images acquired with UAVs and Sentinel satellites.

Acquisition	Maize		Celery		Colza		Cereal *	
	Area [ha]	*HP*_SAVI_	Area [ha]	*HP*_SAVI_	Area [ha]	*HP*_SAVI_	Area [ha]	*HP*_SAVI_
UAV	4.70	42	0.54	55	0.97	38	1.44	32
Sentinel	4.70	58	0.54	71	0.97	37	1.44	76
UAV	2.42	41	0.37	59	0.62	36	0.89	32
Sentinel	2.42	52	0.37	72	0.62	31	0.89	84
UAV	1.31	38	0.17	57	0.36	30	0.55	32
Sentinel	1.31	52	0.17	74	0.36	21	0.55	65
UAV	0.96	49						
Sentinel	0.96	73						

* Buckwheat.

## Data Availability

All the data used in the research was sourced from open source or made own measurements. All data obtained during the study were included in the article.
